# A systematic review of cognitive functioning in early treated adults with phenylketonuria

**DOI:** 10.1186/s13023-018-0893-4

**Published:** 2018-08-30

**Authors:** Denise Leonne Hofman, Claire Louise Champ, Clare Louise Lawton, Mick Henderson, Louise Dye

**Affiliations:** 1 School of Psychology, University of Leeds, Leeds, LS2 9JT UK; 2 Biochemical Genetics, Specialist Laboratory Medicine, St James’s University Hospital, Block 46, Leeds, LS9 7TF UK

**Keywords:** Phenylketonuria, Cognitive function, Attention, Processing speed, Executive function, Motor skills

## Abstract

**Background:**

Even though early dietary management of phenylketonuria (PKU) successfully prevents severe neurological impairments, deficits in cognitive functioning are still observed. These deficits are believed to be the result of elevated levels of phenylalanine throughout life. Research on cognitive functioning in adults with PKU (AwPKU) often focuses on domains shown to be compromised in children with PKU, such as attention and executive functions, whereas other cognitive domains have received less attention. This systematic review aimed to provide an overview of cognitive functioning across domains examined in early treated (ET) AwPKU.

**Methods:**

A systematic search was performed in Ovid MEDLINE(R), PsycINFO, Web of Science, Cochrane, Scopus, Embase, ScienceDirect, and PubMed for observational studies on cognitive performance in ET AwPKU.

**Results:**

Twenty-two peer-reviewed publications, reporting on outcomes from 16 studies were reviewed. Collectively, the results most consistently showed deficits in vigilance, working memory and motor skills. Deficits in other cognitive domains were less consistently observed or were understudied. Furthermore, despite reports of several associations between cognitive performance and phenylalanine (Phe) levels throughout life the relationship remains unclear. Inconsistencies in findings across studies could be explained by the highly heterogeneous nature of study samples, resulting in large inter- and intra-variability in Phe levels, as well as the use of a variety of tests across cognitive domains, which differ in sensitivity. The long-term cognitive outcomes of early and continuous management of PKU remain unclear.

**Conclusions:**

To better understand the development of cognitive deficits in ET AwPKU, future research would benefit from 1) (inter)national multicentre-studies; 2) more homogeneous study samples; 3) the inclusion of other nutritional measures that might influence cognitive functioning (e.g. Phe fluctuations, Phe:Tyrosine ratio and micronutrients such as vitamin B12); and 4) careful selection of appropriate cognitive tests.

**Electronic supplementary material:**

The online version of this article (10.1186/s13023-018-0893-4) contains supplementary material, which is available to authorized users.

## Background

Phenylketonuria (PKU) is a rare (on average 1 in 10.000–12.000 live births in Western Europe) inborn error of metabolism. It is characterised by reduced activity of the hepatic enzyme phenylalanine hydroxylase (PAH), caused by mutations in the encoding gene [1]. To date, around 1044 PAH-gene variants have been documented [2]. PAH is responsible for the conversion of phenylalanine (Phe) to tyrosine (Tyr). Reduced PAH activity results in elevated Phe levels, decreased Tyr levels and an altered Phe:Tyr ratio in individuals with PKU [1]. When left untreated, PKU can cause severe and irreversible neurological impairments [3].

Since its discovery, research into PKU has improved diagnosis and management of the disorder immensely. Patients are diagnosed via new-born screening [4] and, generally, treatment is started as early as possible. Treatment is aimed at keeping Phe levels low [5], but guidelines (target phenylalanine levels) vary between countries [6]. Despite developments of novel treatment strategies i.e. Sapropterin dihydrochloride (Kuvan) and Pegvaliase (Palynzig), the conventional treatment for PKU is still a diet low in protein supplemented with mixtures of free amino acids (other than Phe), vitamins, minerals, trace elements, and essential fatty acids lacking in the low-protein diet [7].

With treatment, severe cognitive impairments are prevented [1]. Nonetheless, deficits in cognitive functioning in PKU patients are still observed. In childhood, deficits are mainly observed in executive functions (EF), such as working memory (WM) and reasoning/planning, attention, and processing speed [8, 9]. In adults, similar deficits have been reported [10]. However, the majority of research has focussed on these specific cognitive domains, whereas other cognitive functions have received less attention. There is a lack of a comprehensive and systematic overview of cognitive functioning across different cognitive domains in early treated adults with PKU (ET AwPKU) assessing the effectiveness of conventional treatment strategies [11].

There is some debate on the specific neuropsychological mechanism(s) responsible for the observed cognitive deficits in PKU, but the general belief is that these deficits are related to patients’ Phe-levels at several stages throughout life (e.g. concurrent Phe levels, lifetime Phe levels, variation in Phe levels, altered Phe:Tyr ratio) [12]. Two theories on the mechanism of action of the disturbed Phe metabolism in PKU have been developed. The first suggests that, because Phe competes with other Large Neutral Amino Acids (LNAA; e.g. Tryptophan (Trp) and Tyr) for transport across the blood-brain barrier (BBB), high levels of Phe saturate the LNAA-transporters. As a result, PKU patients often present with lower brain concentrations of other LNAA and important neurotransmitters serotonin, norepinephrine and dopamine [13, 14], known to be involved in cognitive functioning [15]. Furthermore, it has been suggested that high brain Phe concentrations cause neurotoxicity, which is thought to interfere with cerebral protein synthesis, increase myelin turnover, and inhibit neurotransmitter synthesis [16]. In addition to uncertainties about the exact mechanism underlying suboptimal cognitive functioning, it is unclear whether observed deficits in EFs are the consequence of reduced speed of processing or whether impairments in speed of processing are the consequence of deficits of EF [17].

The overall management of PKU is complex, not only requiring adherence to the PKU diet and Phe-free protein substitute but also requiring regular collection of blood samples, recording of food intake and regular visits to the metabolic clinic [18]. Adherence to the diet and protein substitutes is thought to be especially crucial during the early childhood years since research has shown that cognitive outcomes are closely related to the control of blood phenylalanine levels in this period of life [19, 20], and should be maintained through adulthood to protect from neuropsychological dysfunction [21–24]. However, the strict low-protein diet imposes a burden on patients and their families and has been associated with dietary non-adherence, especially in adolescents and young adults [22, 25–28]. Various metabolic centres have reported increased loss to follow-up and decreased adherence to dietary recommendations when patients grow older [27, 29, 30]. As a consequence, and because ‘diet for life’ is still relatively recent advice [31], the majority of ET AwPKU that have participated in research have discontinued their diet and Phe-free protein substitutes at some point in their lives. Thus very few people with PKU will truly be early and continuously treated, and the impact of such treatment breaks on cognitive function is not known.

This systematic review aims to provide a clear overview of cognitive functioning in ET AwPKU by addressing the following questions: (1) Which cognitive domains are affected in ET AwPKU; (2) How are cognitive outcomes across different domains related to concurrent and lifetime Phe levels in ET AwPKU; and (3) are there any differences in cognitive performance between early and continuously treated (ECT) AwPKU and ET AwPKU who discontinued their diet and/or Phe-free protein substitutes at some point?

## Methods

This systematic review followed the preferred reporting items for systematic reviews and meta-analyses (PRISMA) 2009 checklist and is registered in PROSPERO. The registration number is CRD42016043706 [14].

### Search strategy and search terms

Searches of electronic databases were carried out on 31 July 2017. This search was updated on 2 March 2018 and again on 18 June 2018. Databases searched were Ovid MEDLINE(R), PsycINFO, Web of Science, Cochrane, Scopus, Embase, ScienceDirect, and PubMed 1953 to June 2018. The following search terms were used: (‘phenylketonuria’ OR ‘PKU’) AND (‘cogniti*’ OR ‘memory’ OR ‘attention’ OR ‘visual-spatial’ OR ‘visuo-spatial’ OR ‘recall’ OR ‘recognition’ OR ‘problem solving’ OR ‘reaction time’ OR ‘vigilance’ OR ‘executive function*’ OR ‘reasoning’ OR ‘psychomotor’ OR ‘motor’ OR ‘processing’ OR ‘planning’ OR ‘verbal fluency’ OR ‘inhibit*’).

Furthermore, the reference lists of existing reviews and identified articles were examined individually to supplement the electronic search. A total of 10,803 citations were screened against inclusion and exclusion criteria.

### Inclusion and exclusion criteria

This review was limited to articles published in peer-reviewed journals in English, Dutch or German. Case reports, abstracts and conference proceedings were not included. Papers were included or excluded in this review using the following criteria.

#### Participants

Studies of ET AwPKU aged 18 years and over of either gender were included. As treatment guidelines vary worldwide, age at the start of treatment for the ET AwPKU sample of each paper was included in the data extraction, where available. Animal studies were excluded. Studies where results of ET AwPKU were not reported separately (e.g. papers reporting combined outcomes of ET adolescent and adult PKU patients) were excluded from this review.

#### Intervention

Papers reporting on a sample of ET AwPKU patients who had been treated with the conventional low-protein diet with Phe-free protein substitutes were included. Studies reporting on cognitive outcomes in ET AwPKU as a result of (an acute) manipulation of Phe-levels or additional supplementation with Tyr, or vitamins and minerals were excluded. Finally, as this systematic review aims to give a clear overview of the efficacy of early treatment on cognitive outcomes in adulthood, interventions with new treatments such as Sapropterin dihydrochloride (Kuvan) and Pegvaliase (Palynzig), which were not available when the ET AwPKU commenced their treatment, were excluded.

#### Control(s)

Research including a healthy control group or a comparator group (e.g. diabetic patients, autistic patients) was included. Papers without a specific control group (e.g. comparison to standardized or normative data) were also included.

#### Outcome measures

Studies including any objective measure of cognitive performance were included. Metabolic outcomes (e.g. concurrent Phe levels) were not a requirement for inclusion but were considered where available.

### Design

Observational studies (i.e. cross-sectional, cohort, case-control and longitudinal studies) were included in this systematic review.

### Study selection process

**Fig. 1:**
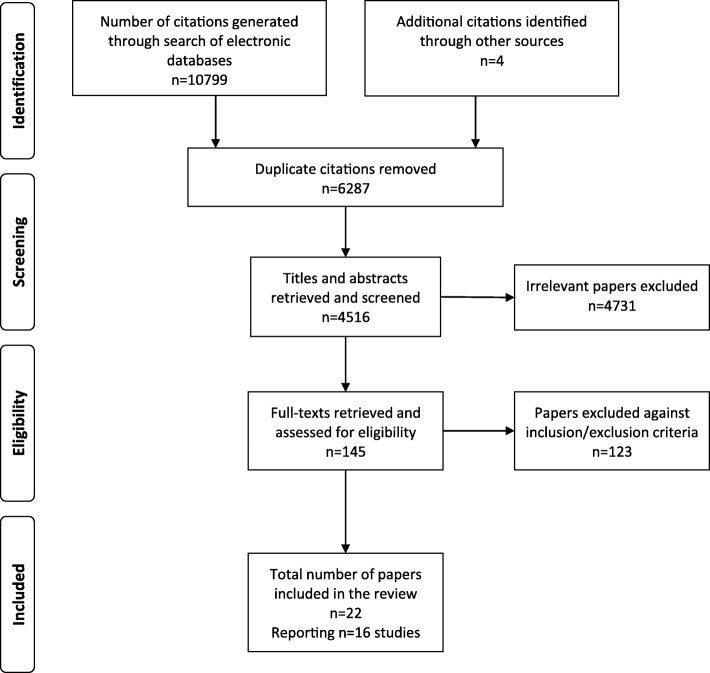
Flow diagram of the study selection process

The literature search yielded a total of 10,803 citations. Following removal of 6287 duplicates, a total of 4516 citations were retrieved for possible inclusion in the review. The titles and abstracts of these citations were screened by one reviewer (DH) to remove obviously irrelevant reports (*n* = 4371), resulting in retention of 145 papers. Another reviewer (CC) independently screened, at random, 5% of the titles and abstracts to establish agreement about the inclusion and exclusion of studies. The inter-rater agreement was 95%, and any disagreements during this process were resolved by discussion, and a consensus decision was reached. The full-text versions of the remaining 145 articles were retrieved and examined for eligibility based on the inclusion criteria, and authors were contacted to clarify any missing information. Inter-rater agreement was 100%. As a result of the screening process, a further 123 articles were excluded. A total of 16 studies reported in the remaining 22 articles were included in the review (see Fig. 1).

### Quality assessment

The quality of all included papers was assessed using the ‘quality assessment tool for reviewing studies with diverse design’ (QATSDD) [32]. Two reviewers (DH and CC) independently awarded each research paper quality scores by assessing each QATSDD criterion (for example ‘Description of procedure for data collection’) on a 4-point scale from 0 to 3 (0 = the criterion is not at all described, 1 = described to some extent, 2 = moderately described and 3 = described in full). The sum of scores of all relevant QATSDD criteria reflects the overall quality of each paper. The scores, expressed as a percentage of the maximum possible score of 42, are included in the data extraction table (Additional file 1: Table S1).

Quality ratings ranged from 35.7 to 59.5% of the maximum score and overall average quality was rated at 48.3%. Papers scored particularly low with respect to reporting of statistics: there was no clear evi.dence of sample size considered in terms of analysis, justification for analytical method selected or assessment of reliability of the analytical process across publications. Publications scored particularly high on the following criteria: explicit theoretical framework, statement of aims/objectives, and description of procedure for data collection. Finally, whilst most papers had strong discussions in terms of interpretation and implications of the data, they lacked a critical discussion of the strengths and weaknesses of the studies reported.

### Data extraction

The Cochrane data extraction form was modified for the purposes of this review. Data were extracted into the standardised form by one researcher (DH), and authors were contacted when insufficient information was provided in the published paper. Half (50%) of these articles were then double data extracted by another researcher (CC). Any disagreements were resolved by discussion, and a consensus decision was reached.

## Results

### Selected studies

Twenty-two articles reporting on outcomes from 16 observational studies assessing cognitive functioning in ET AwPKU were included in this review.

Fourteen studies included healthy controls, often matched for gender and age, and (less often) IQ and socio-economic status. The two remaining studies compared the performance of ET AwPKU on cognitive tasks to either standardized [33] or normative data [34].

Four studies reported on a group of in ET AwPKU who had discontinued their diet [35–38], three of these also included ET AwPKU who were on diet but reported results for on- and off-diet patients separately [36–38]. Furthermore, four studies described their sample of AwPKU as early and continuously treated (ECT) [36, 39–45]. However, the upper range of Phe levels at the time of testing of all ECT AwPKU samples exceeded upper target treatment levels. All other research included a mixed sample of both on-diet ET AwPKU and ET AwPKU who were either off-diet or following a relaxed diet in their study samples.

Seven publications compared effects of high versus low Phe levels [33, 34, 40, 46–49]. However, all of these studies used different cut-off Phe levels for their high and low Phe groups: Bik-Multanowski et al. [34] compared cognitive performance of ET AwPKU with concurrent levels of ≤720 μmol/L and > 720 μmol/L; Brumm et al. [33] used cut-off Phe levels of < 1000 μmol/L and > 1000 μmol/L at the time of testing; Jahja et al. [40] compared effects of concurrent, childhood, adolescent and lifetime Phe by comparing low and high Phe groups according to the most frequently used upper target treatment level during childhood, 360 μmol/L (low: < 360 μmol/L, high: ≥360 μmol/L); Bartus et al. [48], de Felice et al. [47] and Nardecchia et al. [49] compared cognitive functioning of patients with Phe levels below and above 600 μmol/L, a frequently used upper target treatment level during adolescence and adulthood [50]; additionally, Bartus et al. [48] compared cognitive task performance of ET AwPKU with average childhood (0–12 years) Phe below and above 360μmo/L; and, finally, Romani et al. [46] divided their sample into two equally large subgroups based on their adulthood Phe levels (low: < 650 μmol/L, high: > 950 μmol/L), noting that their ET AwPKU group with good metabolic control (low Phe group) had adulthood Phe levels close to current treatment guidelines in the UK (< 700 μmol/L [51]).

The majority of publications (18 reporting results of 14 different studies) looked at correlations between cognitive performance and Phe levels during various periods and at various points throughout life.

Finally, three studies reported on a longer-term follow-up study of ET AwPKU [42, 49, 52]. Two of these compared cognitive outcomes during childhood with cognitive outcomes in the same sample in adulthood [42, 49]. The third followed ET AwPKU over a 5-year period [52].

Included studies, with details of the cognitive tasks and metabolic measures utilised, as well as the reported results are summarised in (Additional file 1: Table S1). Table 1 summarises impairments observed in outcome measures of cognitive functioning, and Additional file 2: Table S2 provides reported correlations between Phe and Tyr levels across the life-span and outcome measures cognitive function. Finally, Table 2 provides an overview of different tasks used across different cognitive domains in the studies included in this review. It shows the frequency of use of each of the tasks across all included studies, as well as their sensitivity in ET AwPKU.

**Table 1:** Overview of impairments reported in outcome measures of cognitive functioning in ET AwPKU across studies

	Discontinued Treatment (Off-Diet)				Mixed Sample (On Diet, Relaxed Diet, Off-Diet)									On-Diet (* = Continuously Treated)							Total
Reference [#]	[37]	[36]	[38]	[35]	[48]	[34]	[33]	[47]	[49]	[11, 17, 46]	[55]	[56]	[52]	[37]	[45]	[39]	[38]	[40, 41, 43]	[42]	[44]	
*N (ET AwPKU)*	*24*	*25*	*56*	*12*	*46*	*49*	*24*	*38*	*14*	*37*	*57*	*25*	*57*	*23*	20*	25*	*21*	55–57*	21*	9*	
Attention & processing speed																					
Attentional capacity	0/1	2/2^e^				0/1	3/4			4/10^d^	1/1			0/1	1/1	1/2^d^					*11/20* ^*k*^
Vigilance/focus	1/1					1/1	0/1			1/1			1/1^b^	1/1	1/1			2/2			*7/8* ^*k*^
Processing speed			1/1^a^	2/2	0/2	0/1	0/2			0/1		1/1	1/1^b^				0/1			0/1	*5/12* ^*k*^
Executive functions																					
Complex EF		0/1		1/2	3/3		1/6		3/13	3/6		0/1			0/2	0/1			1/1^f^		*12/35* ^*k*^
Inhibitory control		1/2^d^				1/1^j^	1/2			0/3						0/2		2/2	0/4	0/1	*5/15* ^*k*^
Working memory	0/1	2/2^e^		0/2	1/1	2/2	2/2			2/3				0/1	1/1	1/2^d^		4/6^g^			*14/20* ^*k*^
Verbal fluency				0/2			2/2			1/2^h^					1/1						*4/8*
Language																					
Basic language skills: semantic processing		0/1		0/1			1/2	0/9		1/5^d^						0/1					*2/18* ^*k*^
Complex language skills							0/2	7/21		3/7					0/1						*10/31*
Memory & learning				0/1																	
Immediate recall: verbal or visual				0/3			2/2^c^			0/3											*2/8*
Delayed recall: verbal or visual				0/2			3/3		0/1	0/3					0/2						*3/11*
Recognition: verbal or visual				0/1			1/1^c^			0/1					0/2						*1/5*
Motor skills					1/1		0/2			2/2	3/4								1/1^f^		*7/10*
Social-cognitive abilities																		2/2^j^			*2/2*
Visual-spatial abilities							1/4		0/1			1/1			0/1						*2/7*

^a^only for off-diet ET AwPKU; ^b^ only for older (> 32) ET AwPKU; ^c^ Verbal memory/learning only; ^d^ significant differences observed in RT, not accuracy; ^e^ accuracy: different to controls, RT only different from on-diet ET AwPKU; ^f^ high-high PKU group worse performance than low-high PKU group (no controls included in analysis); ^g^ differences mainly observed in RT (only 1 measure of accuracy sign. different); ^h^ impairments in semantic, not letter fluency; ^i^ 1/2 disappeared when including age as covariate; no impairments observed when including IQ as a covariate; ^j^ only difference between < 720 and > 720 umol/L (no normative data available); ^k^ where off-diet and on-diet ET AwPKU reported separately, the total represents sum of outcome measures per paper, not row

**Table 2:** Overview of tasks used to assess cognitive functioning in ET AwPKU across different cognitive domains

Cognitive domain	Task^a^	Sensitivity^b^
*Attention and processing speed*		
Attentional capacity	California Verbal Learning Test (CVLT) – List A trial 1 [33]	1/1
	Choice Reaction Time [11, 34]	1/2^c^
	Conjoined Search [11]	1/1^c^
	Detection with Distractors [11]	0/1
	Digit Span Forward (WAIS-R) [33]	0/1
	Feature Search [11]	1/1^c^
	n-back (0-back and 1-back trials) [36]	1/1^c^
	Stroop colour [11, 33]	2/2^c^
	Stroop word [33]	0/1
	Telephone Search Test (TEA) [45]	1/1
	Video tracking [55]	1/1
		***9/13***
Vigilance/focus	Continuous Performance Test (CPT) – omission errors [33]	0/1
	Rapid Visual Processing [11, 34]	2/2
	Sustained Attention Dots (ANT) [40] Dot Pattern Exercise (SVAT) [37]	2/2
	Telephone Search Test with Counting (TEA) [45]	1/1
	Test d2 [52]	1/1^d^
		***6/7***
Processing speed	CPT – response rate [33]	0/1
	Motor Screening Test – latency [48]	0/1
	Saccadic Latency [38]	1/1^e^
	Simple Detection [11] Simple Reaction Time (CANTAB) [34] Finger Motor Speed Exercise (SVAT) [37]	0/3
	Stockings of Cambridge – initial thinking time [48]	0/1
	Trail Making Test-A [1, 2, 33, 52] Attention Diagnostic Method [56]	2/3
	WAIS-III (Processing Speed Index) [35]	1/1^e^
		***4/11***
*Executive functions*		
Complex executive functions	Brixton Test [45]	0/1
	Elithorn Perceptual Maze Test [49]	1/1
	Object alternation learning [36]	0/1
	Set Shifting Visual (ANT) [42]	1/1^f^
	Six Elements Test [45]	0/1
	Spatial Working Memory – Strategy [48]	1/1
	Stockings of Cambridge [48]	1/1
	Tower of Hanoi [11] Tower of London [49]	1/2
	Trail Making Test (TMT) B-A [11, 33, 35]	0/3
	WAIS-III (Perceptual Organisation Index) [35]	1/1^e^
	Wisconsin Card Sorting Test (WCST) [11, 33, 49, 56]	3/4
		***9/17***
Inhibitory control	CPT [33]	1/1
	Flanker [36, 42]	1/2^c^
	Go-nogo [44]	0/1
	Set Shifting Visual (ANT) [42]	0/1
	Stop Signal Task [34]	1/1
	Stroop (interference) [11, 33]	0/2
	Sustained Attention Dots (ANT) [40]	1/1
		***4/9***
Working memory	Corsi Block Tapping Test [11]	0/1
	CVLT – perseverative error [33]	1/1
	Digit Span – backward [11, 33]	2/2
	Feature Integration (ANT) [40]	1/1
	Letter Pattern Exercise (SVAT) [37]	0/1
	Memory Search 2 Dimensional (ANT) [40]	1/1
	n-back (2-back trials) [36]	1/1^c^
	Non-word Repetition [11]	1/1
	Self-Ordered Pointing test [45]	1/1
	Spatial Span [34]	1/1
	Spatial Working Memory [34, 48]	2/2
	Visuo-Spatial Sequencing (ANT) [40]	1/1
	WAIS-III (Working Memory Index) [35]	0/1
		***12/15***
Verbal fluency	Animal naming [11, 33]	2/2
	Controlled Oral Word Association Test [33, 35]	1/2
	Letter fluency [11, 45]	1/2
		***4/6***
*Language*		
Basic language skills: semantic processing	Boston Naming Test [33] Picture naming [11]	1/2
	Blocked cyclic naming [47]	0/1
	Emotional Prosody Discrimination test [47]	0/1
	Hayling Sentence Completion Test – Part A [47]	0/1
	Narrative production: Recalling the Cinderella story [47]	0/1
	Non-emotional Prosody Discrimination test [47]	0/1
	Peabody Picture Vocabulary Test – Revised (PPVT-R) [33]	0/1
	Word reading [11]	1/1^c^
	Word spelling [11]	0/1
		***2/10***
Complex language skills	Appreciation of Humour test [47]	0/1
	Blocked cyclic naming – semantic Inference [47]	0/1
	Comprehension of Inferred Meaning test [47]	1/1^c^
	Conflicting prosody – attend to prosody test [47]	0/1
	Hayling Sentence Completion Test – Part B [45, 47]	1/2
	Metaphor Picture test [47]	1/1^c^
	Naming: semantic inference [11]	0/1
	Narrative production: Recalling the Cinderella story [47]	1/1
	Non-word reading [11]	1/1^c^
	Non-word spelling [11]	0/1
	Phoneme deletion [11]	0/1
	Similarities (WAIS-R/WASI) [11, 33]	1/2
	Spoonerisms [11]	0/1
	Vocabulary (WAIS-R/WASI) [11, 33]	0/2
	WAIS-III (Verbal Comprehension Index) [35]	0/1
	Perceptual judgement task [36]	0/1
		***6/19***
*Memory and learning*		
Immediate recall: verbal / visual	CVLT – List A trial 5 and trials 1–5 [33]	1/1
	Paired Associates Verbal Learning – trial 1–5 [11]	0/1
	Paired Associates Visual Learning [11]	0/1
	Rey Auditory Verbal Learning Test (RAVLT) – Trial A1-A5 [11]	0/1
	ROCFT – Immediate recall [49]	0/1
	WMS-III [35]	0/1
		***1/6***
Delayed recall: verbal / visual	CVLT – Sort delayed recall and Long delayed recall [33]	1/1
	Paired Associates Verbal Learning – delayed [11]	0/1
	RAVLT – Delayed recall [11, 45] and retention [11]	0/2
	ROCFT – Delayed recall [33, 45]	0/2
	WMS-III [35]	0/1
		***1/7***
Recognition: verbal / visual	CVLT – Recognition memory [33]	0/1
	Delayed Matching to a Sample [11]	0/1
	RAVLT – Recognition [45]	0/1
	ROCFT – Recognition [45]	0/1
	WMS-III [35]	0/1
		***0/5***
*Motor skills*	Digit Symbol (Substitution) Task [11, 33]	1/2
	Grooved Pegboard [11, 33]	1/2
	MLS [55]	1/1
	Motor Screening Test – errors [48]	1/1
	Pursuit (ANT) [42]	1/1^f^
		***5/6***
*Social-cognitive abilities*	Face Recognition (ANT) [41]	1/1^g^
	Faux-Pas Recognition Test [41]	1/1^h^
	Identification of Facial Emotions (ANT) [41]	1/1^g^
	Reading the Mind in the Eyes Task [41]	1/1^h^
		***4/4***
*Visual-spatial abilities*	Block design (WAIS-R) [33]	0/1
	Picture arrangement (WAIS-R) [33]	0/1
	Picture completion (WAIS-R) [33]	0/1
	ROCFT – with copy/initial copy [33, 45, 49, 56]	2/4
		***2/7***

^a^ Number of observed impairments in task performance in ET AwPKU / frequency of us; ^b^ (−sub measure) if task relates to multiple cognitive domains); ^c^ Compared to controls, ET AwPKU differed in speed (slower), not accuracy; ^d^ Only for older (>32) ET AwPKU;

^e^ Only for off-diet ET AwPKU; ^f^ High-high ET AwPKU group worse performance than low-high PKU group (no controls included in analysis); ^g^ Effect disappeared after adding age as a covariate; ^h^ Effect disappeared after including IQ as a covariate

### Cognitive outcomes in ET AwPKU: Overview of reported results

The following section provides an overview of cognitive outcomes in ET AwPKU. Where possible, outcomes in adulthood are compared with outcomes in the same sample during childhood [42, 49].

As can be seen in (Additional file 1: Tables S1) and Table 2, a large number of different cognitive tasks were used, spanning various cognitive domains. Furthermore, some discrepancy between papers exists with regards to the domains that cognitive tasks are ascribed to. For the purpose of this review, cognitive outcomes are categorised according to their cognitive domains. There are many different conceptualisations regarding how different cognitive tasks associate with one another and with particular cognitive domains. The framework used for the current review was adapted from a commonly used approach to understanding and measuring cognitive domains [53]. For a description of cognitive domains, subdomains and examples of tests reflecting each domain as applied to the studies reported in this review, see Galioto et al. [54]. Note, however, that Galioto et al. [54] describe verbal fluency as a function of language, whereas this review follows Lezak et al. [53]‘s original framework, classifying it as an EF. Additional file 1: Table S1 summarises cognitive outcomes as reported in the papers included in this review. In Tables 1, 2 and Additional file 2: Table S2, outcomes have been re-categorised in line with the framework used here.

### Attention and processing speed

#### Attentional capacity

Healthy controls outperformed ET AwPKU on the majority of measures of attentional capacity used across several studies included in this review [11, 33, 36, 45]. However, it was found that ET AwPKU were often slower, but not less accurate, than controls [11, 36]. Furthermore, Channon et al. [36] observed differences in accuracy between off- and on-diet ET AwPKU, with the off-diet group making more errors compared to the on-diet group. Using an aggregate score for performance on attention tasks included in their study, Romani et al. [46] reported that the ET AwPKU with low adult Phe levels significantly outperformed the high-Phe group. Bik-Multanowski et al. [34] and Brumm et al. [33] found no differences in performance ET AwPKU with high compared to low concurrent Phe levels.

The relationship between performance on tasks reflecting attentional capacity and measures of metabolic control was assessed in seven studies. Only two of these reported a relationship between concurrent Phe and measures of attentional capacity [36, 55]. However, the observed correlations were not in the expected direction, suggesting that attentional capacity was better with higher concurrent levels of Phe. Several papers reported significant correlations with metabolic control during childhood [33, 36, 46], adulthood [17, 45] as well as throughout life [46], with the majority (*n* = 10/11, see Additional file 2: Table S2) suggesting lower Phe levels were associated with better task performance. However, no correlations between adolescent Phe levels and attentional capacity were reported. Furthermore, the correlations observed by Channon et al. [36] were limited to measures of speed, with no correlations for accuracy.

#### Vigilance/focus

Compared to healthy controls, ET AwPKU have consistently been found to show impairment on measures of vigilance/focus [11, 37, 40, 45, 52]. In one study, however, this impairment was only observed in older (> 32 years old) ET AwPKU [52]. Brumm et al. [33] reported no group deficit on a continuous performance task (CPT) when comparing number of omission errors of ET AwPKU with normative data, but did find that ET AwPKU with high concurrent Phe performed significantly worse than those with low concurrent Phe. This is in line with results reported by Bik-Multanowski et al. [34] and Romani et al. [46], although observed differences in performance of the low and high Phe groups in the latter study failed to reach significance.

Observed associations between measures of metabolic control and vigilance in ET AwPKU are somewhat inconsistent but suggest childhood Phe levels are not related to vigilance in ET AwPKU, whereas significant negative correlations with adult Phe have been found. Inconsistent results have been reported for concurrent, adolescent and lifetime Phe levels. Jahja et al. [42] and Romani et al. [46] reported significant correlations between concurrent Phe and measures of vigilance, whereas Brumm et al. [33] did not. Romani et al. [46] also reported a significant association between vigilance and metabolic control during adolescence. However, this was not observed by Weglage et al. [52]. Finally, Romani et al. [46] found a significant correlation between an aggregated score of measures of vigilance and lifetime Phe, whereas Jahja et al. [42] reported no significant associations between the two.

#### Processing speed

It has been suggested that observed cognitive deficits in ET AwPKU could be due to a deficit in information processing in these patients. It is not uncommon for ET AwPKU to be slower, but not less accurate on various measures spanning different cognitive domains. Romani et al. [17] investigated processing speed in ET AwPKU. Their results suggest that ET AwPKU do not suffer from an overarching deficit in speed of processing, but rather that reduced speed of performance on tasks across multiple cognitive domains could be the result of slower or more cautious executive decision-making processes [17].

In line with their findings, performance of ET AwPKU on ‘pure’ processing speed outcome measures, such as simple reaction time, was not generally impaired in the studies included in this review. Compared to controls, ET AwPKU demonstrated slower reaction times on approximately half of the processing speed measures reported in studies included in this review [35, 38, 52, 56]. In two of these studies, these deficits were observed in a group of ET AwPKU who had discontinued dietary treatment [35, 38]. In another study, the impairment in information processing was only found for older (> 32 years) ET AwPKU [52]. However, four of the studies included in this review reported no impairments in performance on measures of processing speed in either on or off-diet ET AwPKU [11, 33, 37, 48]. When comparing groups of ET AwPKU with different levels of metabolic control, Brumm et al. [33] reported that ET AwPKU with high concurrent Phe levels were significantly slower than those with low concurrent Phe levels, whereas Bik-Multanowski et al. [34] and Bartus et al. [48] found no differences between patients with good versus poor concurrent and childhood (between 0 and 12 years) metabolic control.

Five studies investigated associations between simple measures of processing speed and measures of metabolic control. Brumm et al. [33] and Bartus et al. [48] observed no correlations, whereas Weglage et al. [52] reported negative correlations with Phe levels during childhood, adolescence and young adulthood. Furthermore, two studies reported a relationship between speed of processing and concurrent Phe levels, but the direction was inconsistent: one study reported a negative relationship [38] while the other reported a positive relationship [56]. Significant correlations were generally more frequently observed with measures of speed compared with measures of accuracy.

### Executive functions

#### Complex executive functions

Although reasoning and planning, flexibility (set-shifting/switching), organisation, monitoring and rule finding are separate executive functions (EF), several of the cognitive tasks used in the studies reported here concurrently engage more than one EF and are often reported as measures of complex EF, higher order EF, or “multi-tasking”. Reported findings across studies suggest a contrast between performance on tasks that require different levels of planning/reasoning and flexibility, with deficits in ET AwPKU being more pronounced in tasks requiring more planning/reasoning and flexibility. For example, deficits in performance on the Wisconsin Card Sorting Test (WCST) were reported by Brumm et al. [33], Nardecchia et al. [49] and Palermo et al. [11], but not by Ris et al. [56]. Furthermore, Bartus et al. [48] reported that controls outperformed ET AwPKU on measures of problem solving (Stockings of Cambridge of the Cambridge Neuropsychological Test Automated Battery (CANTAB)) and strategy (Spatial Working Memory (CANTAB)), whereas Channon et al. [45] and Nardecchia et al. [49] did not observe any deficits in performance on the Brixton task or Elithorn Perceptual Maze Test respectively. Some of the reported impairments in complex EF were only observed for ET AwPKU with poor metabolic control throughout childhood [42, 48] or off-diet ET AwPKU [35]. However, although ET AwPKU with lower concurrent Phe-levels showed better performance on complex EF tasks, none of the studies reported significant differences between ET AwPKU with good versus poor concurrent metabolic control [33, 46, 48].

Relationships with metabolic control throughout life and complex EF were observed, but better metabolic control during adolescence seems to be the strongest indicator of better complex EF during adulthood [46, 49]. Reported correlations between concurrent Phe and complex EF were not in the expected direction, suggesting ET AwPKU with higher concurrent levels of Phe performed better on complex EF tasks than those with better metabolic control at the time of testing [35].

#### Inhibitory control

The majority of the studies that included measures of inhibitory control did not reveal any significant impairments in inhibition in ET AwPKU compared to controls [11, 40, 42, 44], although the PKU group tended to be slower, not less accurate, than the control group in one of the studies included in this review [36]. The PKU-COBESO study was the only study to report ET AwPKU were both significantly less accurate and slower compared to controls [40]. Moyle et al. [44] observed a similar trend in a smaller sample of ET AwPKU but failed to find any significant differences. Based on normative data available for measures included in their study, Brumm et al. [33] reported that ET AwPKU performed below expectation (see Additional file 1: Table S1) on several (CPT, Digit Span backwards and WCST), but not all (Stroop, Trail Making Task part B), measures of inhibitory control. However, they observed no significant differences in performance between ET AwPKU with good and poor concurrent metabolic control on any of the tasks. Similarly, a recent study found no significant differences in task performance between ET AwPKU with low and high concurrent Phe levels [46]. In contrast, Bik-Multanowski et al. [34] reported significant differences in performance on the CANTAB Stop-Signal Task between ET AwPKU with good and poor metabolic control, with the ET AwPKU with poor metabolic control showing worse performance.

After splitting their ET AwPKU sample into high and low Phe groups, Jahja et al. [40] reported that, compared to controls, only ET AwPKU with high lifetime Phe levels were slower and less accurate on an inhibitory control task. Furthermore, their results showed that concurrent Phe was positively associated with reaction times, but no correlations between childhood, adolescent, adult or lifetime Phe levels and accuracy or speed were found. Romani et al. [46] observed no correlations between measures of inhibition and any of the measures of metabolic control included in their research.

#### Working memory

Studies investigating performance of ET AwPKU on WM or short-term memory (STM) tasks showed contradictory findings [11, 33–37, 40, 45, 48].

In terms of accuracy, the majority of studies reported that ET AwPKU made significantly more errors compared to controls or normative data [11, 33, 34, 42, 45, 48]. In contrast, the remaining three studies, two of which included off-diet ET AwPKU, did not find significant differences in accuracy on WM tasks between ET AwPKU and healthy controls [35–37]. Even though they did not observe any differences between ET AwPKU and controls, Channon et al. [36] reported that off-diet ET AwPKU were significantly less accurate on the n-back task than on-diet ET AwPKU. Again, roughly half of the papers reporting measures of speed found that both on-diet and off-diet ET AwPKU were significantly slower than healthy controls [36, 40]. Jahja et al. [40] observed a significantly greater decline in speed with increasing WM load on two of their measures, whereas other studies did not [35, 36].

When exploring relationships between WM and metabolic control, Channon et al. [45] reported poor performance on WM tasks was related to high concurrent and average recent (year preceding testing) Phe levels as well as elevated Phe levels between the ages of 21 and 28 years. In another study, despite not showing any significant deficits in WM in on-diet and off-diet ET AwPKU, speed on the 2-back task was found to be related to Phe levels between the ages of 13–16 years [36]. In the PKU-COBESO study [40], ET AwPKU were divided into low- and high-Phe groups based on concurrent as well as average childhood, adolescence, adult and lifetime Phe levels. In line with findings of Bik-Multanowski et al. [34], results showed that higher concurrent Phe levels resulted in slower speed on two of the three WM tasks used in this study (Feature Integration (FI) and Memory Search 2-Dimensional (MS2D) of the Amsterdam Neurological Tasks (ANT) battery). Additionally, lifetime Phe levels were positively related to the number of errors made on tasks with a high WM load. Furthermore, analyses revealed that ET AwPKU with high average childhood Phe levels were significantly less accurate than controls on two of three WM tasks (Visuo-Spatial Sequencing (VSS) and FI). They were also significantly less accurate on the FI task compared to ET AwPKU with low childhood Phe levels. Finally, ET AwPKU with high childhood and lifetime Phe levels were found to be significantly slower than controls on the MS2D task. Romani et al. [46] did not find any significant relationships between WM performance and concurrent Phe or averages and variations of childhood, adolescent, adult and lifetime Phe levels, but reported that the group with low concurrent Phe levels outperformed the high-Phe group. Bartus et al. [48] did not find any significant differences in accuracy on the SWM (CANTAB) task between on-diet ET AwPKU and those on a “loose diet”, but did show that ET AwPKU with better metabolic control during childhood made less errors than those with poorer control.

There does not seem to be a clear association between measures of WM and measures of metabolic control: the majority of studies observed no relationships, with the exception of some correlations found with concurrent Phe and Phe at different stages of life (see Additional file 2: Table S2) [33, 36, 40, 45].

#### Verbal fluency

Verbal fluency refers to the ability to orally produce words that either fit into a specific category (category or semantic fluency) or start with a specific letter (letter or phonemic fluency). It has been suggested that language processing is the critical component of verbal fluency [57]. However, because verbal fluency tasks involve a planned, systematic search of the lexicon, they are often regarded as measures of EF [53]. Four studies included in this review assessed verbal fluency in ET AwPKU [11, 33, 35, 45]. Letter fluency was reported to be impaired by Brumm et al. (2004) and Channon et al. [45], but not Palermo et al. [11]. Palermo et al. [11] did, however, find deficits in category fluency, as did Brumm et al. [33]. In contrast, Moyle et al. [35] found no deficits in either category or letter fluency in a small sample of off-diet ET AwPKU. There was no clear evidence for associations between metabolic control and verbal fluency abilities in ET AwPKU.

### Language (semantic processing)

Measures of semantic processing assess comprehension of language as well as speed of retrieval of information [53, 54]. Examples of semantic processing tasks include expressive and receptive vocabulary, expressive naming (spoken language), as well as measures of spelling and reading (orthographic language). Five studies assessed language processing in ET AwPKU. In contrast to Brumm et al. [33], Palermo et al. [11] and de Felice et al. [47] found no deficits on a basic picture naming task. In line with this, apart from a reduction in speed of word reading [11], no issues in basic language skills, including receptive vocabulary, as well as measures of prosody, reading and spelling without inference were observed in ET AwPKU [11, 33, 47]. Performance of ET AwPKU on complex language tasks, requiring EF such as planning, inhibition and reasoning, has been inconsistent (see Table 2). Most studies reported no deficits [33, 35, 36], but impaired performance has been observed on several, but not all, complex language tasks included in two studies [11, 47]. When measures of accuracy and speed have been reported separately, it appears that ET AwPKU are slower but not less accurate on tasks that suggest impaired complex language processing [11, 47].

With respect to the impact of Phe, Romani et al. [46] found significant correlations between a composite measure of the spoken language tasks used in their study (picture and colour naming and both WASI verbal subtests) and fluctuations in Phe as well as overall metabolic control, but not average Phe levels, across the lifespan. No correlations were found between any metabolic measures and performance on tasks assessing orthographic language. Furthermore, ET AwPKU with better metabolic control during adulthood performed better on all language tests, but these differences were only significant for measures of spoken language. However, no significant differences in composite measures of spoken or orthographic language were observed between groups of ET AwPKU with high versus low concurrent Phe levels [46]. Brumm et al. [33] reported that performance on spoken language tasks (expressive naming, expressive vocabulary and receptive vocabulary, but not the similarities subtest of the Wechsler Adult Intelligence Scale-Revised (WAIS-R)) was better in ET AwPKU with better metabolic control at the time of testing and that performance on these measures was negatively correlated with blood Phe levels across the lifespan. De Felice et al. [47] found no associations between measures of metabolic control and any of the language processing measures administered and, moreover, reported no significant differences in performance between ET AwPKU with low versus high average Phe levels.

### Memory and learning

The majority of studies assessing verbal and visual immediate recall, delayed recall, or recognition memory in both on-diet and off-diet ET AwPKU did not report any impairments in ET AwPKU [11, 23, 45, 49]. However, Romani et al. [46] reported that despite not finding any significant differences between ET AwPKU and controls on individual tasks of memory and learning, ET AwPKU seemed to perform marginally worse across tasks when the scores were aggregated [46]. Furthermore, they reported that their lower-Phe group outperformed their higher-Phe group. In contrast, Brumm et al. [33] reported cognitive impairments in immediate, short-term and long-term verbal and visual delayed recall, but did not report any significant differences in memory task performance between ET AwPKU with high or low concurrent Phe levels. In their study, immediate and delayed verbal and visual recall were found to be negatively correlated with median Phe levels between the ages of 5.5 and 6 years [33]. Romani et al. [46] reported negative correlations between aggregated memory and learning scores and concurrent Phe as well as average Phe and variation of Phe levels across the lifespan. Other studies found no correlations between performance on memory tasks and any of the metabolic measures included [23, 45, 49].

### Motor skills

Results of assessments of motor skills are mixed but suggest an impairment in ET AwPKU [11, 33, 42, 48, 55]. Using a battery of 7 tests to assess fine motor abilities, Pietz et al. [55] reported deficits in steadiness (tremor), dexterity and speed, but not visuomotor abilities. None of the observed deficits appeared to correlate significantly with any of their indices of metabolic control. Jahja et al. [42] found that ET AwPKU with low average Phe levels during childhood were better at a motor task that involved continuous monitoring of task performance (following a randomly moving target) than those who had high average childhood Phe levels. They reported significant correlations between task performance and childhood Phe levels [42]. Palermo et al. [11] also observed significant deficits in ET AwPKU on two tasks (Digit Symbol Substitution Task (DSST) and Grooved Pegboard) assessing visuomotor coordination and, using a composite measure, reported that ET AwPKU with low concurrent Phe levels outperformed those with high levels at the time of testing. Furthermore, they reported significant correlations between a composite score of both tasks and concurrent Phe, childhood Phe variation and average levels, and adolescent, adult and lifelong Phe variation as well as overall metabolic control [46]. Using the same two tasks, Brumm et al. [33] did not find any deficits in ET AwPKU but did report that ET AwPKU with low Phe levels at the time of testing outperformed those with high concurrent Phe levels on the DSST. Finally, Bartus et al. [48] reported significant differences in accuracy on the CANTAB Motor Screening Test (MOT) between ET AwPKU and controls, with controls outperforming the ET AwPKU, but did not report any differences between ET AwPKU with good versus poor metabolic control during childhood (0–12 years) or at the time of testing. Both Brumm et al. [33] and Bartus et al. [48] did not find any associations between visuomotor coordination and any of the metabolic outcomes included in their studies.

### Social-cognitive abilities

*“Social cognition involves all mental processes that underlie social interactions and comprises the ability to perceive, to interpret and to respond appropriately to social cues”* ([40], p., 356)*.* Examples of social-cognitive abilities include the ability to recognise faces and identify emotions [41]. Only one study to date has assessed social-cognitive abilities in ET AwPKU [41]. ET AwPKU performed worse than controls on all four tasks included in the research. When controlling for age, impairments in ET AwPKU were only observed on two of the tasks. When IQ was taken into account, no significant differences between ET AwPKU and controls were reported. No significant associations between social-cognitive outcomes and concurrent or lifetime measures of metabolic control were found.

### Visual-spatial abilities

Measures of visual-spatial abilities reflect planning, reasoning, memory and motor skills. Using the ‘with copy’ subtest of the Rey Österrieth Complex Figure Test (ROCFT), two studies reported impairments in a mixed sample of on- and off-diet ET AwPKU [33, 56], whereas two other studies did not [45, 49]. Furthermore, Brumm et al. [33] found no impairments on visual-spatial subtests of the WAIS-R (Block Design, Picture Arrangement and Picture Completion) and no difference in performance between ET AwPKU with high versus low concurrent Phe levels on any of the visual-spatial measures included in their study. They did, however, observe negative correlations between performance on two of the WAIS-R subtests (Block Design and Picture Completion) and median Phe levels between the ages of 5.5–6 and 9.5–10 years. Other studies did not observe any associations between visual-spatial abilities and measures of metabolic control [49, 56].

### Cognitive outcomes in early treated adults with PKU (ET AwPKU): Long-term follow-up

Two studies included in this review were long-term follow-up studies of a cohort of ET AwPKU who participated in research during their childhood: Nardecchia et al. [49] assessed cognitive functioning of 14 ET AwPKU previously examined by Leuzzi et al. [58]. Jahja et al. [42] tested 21 of 69 ET AwPKU (48 of the original sample were lost to follow-up (69%)) who had previously been involved in the study by Huijbregts et al. [59–61]. Both follow-up studies were conducted approximately 14 years after the original research and found that cognitive performance across a range of tests, mainly assessing EF, either remained stable or improved [42, 49]. Nardecchia et al. [49] noted that differences in neuropsychological outcome between ET PKU and controls had become smaller at T2, but had not disappeared entirely. Furthermore, as expected, Phe levels increased with age and results suggest that ET AwPKU who had low childhood Phe and those who had better metabolic control during adolescence had better cognitive outcomes in adulthood [42, 49].

## Discussion

### Summary of findings

#### Cognitive functioning

Cognitive performance of ET AwPKU varied across the different studies and cognitive domains included in this review. In general, impairments in cognitive functioning across domains tended to be observed more on measures of speed than accuracy. ET AwPKU were slower when compared to healthy controls or normative data. However, these speed deficits were rarely observed in tasks of ‘pure’ processing speed (e.g. simple reaction time), apart from in off-diet ET AwPKU [35, 38]. As suggested by Romani et al. [17], these findings could indicate that ET AwPKU may not suffer from a processing speed deficit per se. Reductions in speed of performance across multiple cognitive domains are more likely to be the result of speed-accuracy trade-offs due to slower or more cautious executive decision-making processes.

Compared to healthy controls and normative data, impairments in cognitive performance of ET AwPKU have been most consistently found on tasks of vigilance, WM and motor skills. Furthermore, there is some evidence for deficits in performance on tasks of attentional capacity, verbal fluency, complex language skills, complex EF and inhibitory control. For both complex EF and WM tasks, deficits appear to be more pronounced on tasks which have a higher cognitive load, i.e. requiring more planning/reasoning and flexibility or WM, respectively. Performance on tasks of simple processing speed, memory, visual-spatial abilities, and simple language processing does not seem to be impaired in ET AwPKU. Social-cognitive abilities were reported to be affected in ET AwPKU, but these abilities were only assessed in one of the 15 studies included in this review. Finally, Jahja et al. [42], Nardecha et al. [49] and Weglage et al. [52] reported that overall cognitive performance remained stable or improved over extended periods, despite an observed increase in Phe. This could be due to adequate adherence to treatment after childhood.

#### Impact of metabolic control on cognitive performance

##### Good versus poor metabolic control

Several papers included in this review explored differences in cognitive performance between groups with high versus low levels of Phe at the time of testing, often using different criteria to discriminate the high- and low-Phe groups. Some, but not all, of these studies reported that ET AwPKU with low concurrent Phe levels outperformed ET AwPKU with high concurrent Phe on tasks of selective attention, memory and learning, and semantic language skills. The majority of studies observed a similar pattern for performance on sustained attention tasks as well as motor skills. No differences in performance on visual-spatial measures or measures of complex EF were observed between groups of ET AwPKU with high and low Phe levels at the time of testing. Results from a few studies suggest that ET AwPKU with high Phe levels at the time of testing may have worse inhibitory control than those with low concurrent Phe levels. Finally, some studies suggest that ET AwPKU with high concurrent Phe and those with high childhood-Phe levels are more at risk of developing WM impairments compared to ET AwPKU with low concurrent or childhood Phe, respectively.

##### Associations with metabolic control throughout life

Associations between Phe levels and memory and learning, as well as motor skills, were observed across the lifespan. The relationship appears more robust for visual delayed and recognition memory than measures of verbal memory. Language skills appear to be moderately correlated with childhood Phe levels, which might reflect the fact that language skills are developed during childhood [62]. In contrast, vigilance, complex EF, inhibition and WM were most frequently reported to be correlated with lifetime Phe and Phe later in life (concurrent Phe and Phe during adolescence and adulthood). A possible explanation for this is that these cognitive functions, supported by the prefrontal cortex, are affected by decreased levels of dopamine resulting from poor metabolic control [63, 64]. Limited associations were observed between verbal fluency and concurrent and childhood Phe levels and no associations between Phe and social-cognitive abilities and visual-perceptual abilities were found. Furthermore, limited evidence suggests fluctuations in Phe levels throughout life affect cognitive performance of ET AwPKU. Finally, in studies reporting relationships with Phe for outcome measures of speed and accuracy separately, significant correlations were generally more frequently observed with measures of speed compared to measures of accuracy. Speed-specific associations were predominantly observed with Phe earlier in life (childhood and adolescent Phe). As suggested by Romani et al. [46], speed deficits might be modulated by structural myelin damage caused by suboptimal Phe control early in life.

The vast majority of reported correlations were of moderate strength (see Additional file 1: Table S1) and in the expected direction, such that cognitive performance worsened with an increase in Phe.

### Limitations/ methodological issues

Several factors may have contributed to inconsistent findings across studies in ET AwPKU.

#### Sample

Samples of ET AwPKU are highly heterogeneous: patients are likely to have different PAH-genotypes and will have had varying degrees of dietary adherence throughout life and at the time of testing, leading to inter and intra-individual variability in Phe-levels. Furthermore, some studies included mixed samples of on-diet and off-diet ET AwPKU in the same analysis, whereas others split samples based on their dietary management status. However, no studies clearly defined what was meant by ‘off-diet’, and it is unclear whether the ET AwPKU included followed an omnivorous diet, vegan or vegetarian diet or whether they were still (unconsciously) limiting their protein intake. ET AwPKU doing the latter might suffer from nutritional deficiencies [65] that could affect cognitive functioning (e.g. vitamin B12 [66, 67]) alongside raised Phe. Moreover, although some authors stated that their sample of ET AwPKU were continuously treated, they report concurrent Phe-levels outside of target treatment ranges, suggesting that at least some of their sample were not adherent to dietary recommendations at the time of testing. Therefore, the question remains whether observed cognitive deficits are present in ECT AwPKU. Future research would benefit from the inclusion of additional nutritional measures to better characterise the sample of ET AwPKU and explore the impact of potential nutritional deficiencies on cognitive outcomes. Moreover, to better evaluate the efficacy of current treatments, research should focus on homogeneous samples, or, where this is not possible, include an analysis of carefully characterised subgroups (e.g. on-diet and off-diet).

The inconsistent findings in ET AwPKU in the studies included in this review may be due to issues of sample size. Because PKU is a rare disorder, it is difficult to recruit and retain large samples. Generally, studies of PKU tend to consist of small single centre studies, with a limited number of PKU patients living within study catchment areas. Studies on cognitive performance in ET AwPKU often include a relatively small (< 50 AwPKU) number of participants [10] and are likely to be underpowered. For example, Moyle et al. [35] observed no impairments in cognitive functioning in 12 ET AwPKU who discontinued their treatment during adolescence, whereas Palermo et al. [11] and Jahja et al. [40] reported several deficits in cognitive functioning in relatively well controlled ET AwPKU (*n* = 37 and *n* = 57, respectively). Research in PKU may benefit from more national and international multi-centre collaborations, in order to increase sample size to achieve sufficient power, and address the need to recruit more homogeneous samples.

Furthermore, ET AwPKU who participate in research are likely to be a self-selected sample who are more engaged with their dietary management which could positively bias findings. Deficits in cognitive functioning are likely to be more prevalent and more severe in those who are less adherent to their dietary management, but these patients are likely to be underrepresented in the literature. To illustrate, in the PKU COBESO study, only 21 of the original 68 ET PKU patients took part in the long-term follow-up study [42]. Authors reported that at initial testing, approximately 14 years earlier, this subsample did not differ from controls on any of the cognitive measures, whereas the sample as a whole showed signs of cognitive impairments on several measures. Furthermore, the patients who were lost to follow-up had higher Phe levels at the time of initial testing. This suggests that those patients who were retained for a second test demonstrated better adherence to their dietary management than the ET AwPKU who were lost to follow-up. The percentage of participants who were lost to follow-up in this research (69% of the original sample) is similar to the percentage of AwPKU who were estimated to not access regular clinical therapy in the United States in 2013 (> 70%) [68], suggesting little is known about cognitive functioning in the majority of ET AwPKU. To our knowledge, only a few studies have assessed cognitive performance in a group ET AwPKU who discontinued their diet [35, 37, 38].

#### Cognitive performance testing

As is apparent from Table 2, the studies included in this review used a wide variety of cognitive tests spanning a range of cognitive domains and differing in sensitivity. Besides sample size affecting the power of a study to detect any cognitive deficits, cognitive tests differ in sensitivity. This makes it difficult to compare outcome measures from different studies and draw coherent conclusions. Furthermore, a number of the tests employed in the studies do not necessarily test just one cognitive domain, but rather recruit multiple cognitive functions simultaneously. This can lead to discrepancies in the interpretation of results. For example, the Stroop word and colour subtests are regarded as language skills by Palermo et al. [11] whereas others have reported Stroop to be a measure of attention [33]. Additionally, because they require planning a systematic search of the lexicon, tests of verbal fluency are often believed to reflect EF [69, 70]. However, as these tests tap into the lexicon, one could also argue that performance primarily reflects language skills [57]. In line with the framework used in this review [53], the majority (3/4) of studies that included tests of verbal fluency classified these as a measure of EF. In addition to discrepancies in the interpretation of cognitive test performance, there are also discrepancies in the manner of reporting cognitive outcomes. Most papers report outcomes of speed and accuracy separately, where possible. However, Romani et al. [46] used aggregated scores of cognitive performance on tests attributed to a cognitive domain to explore the association with metabolic control. Limitations of the use of aggregated scores, even if well-constructed, are potential differences in reliability and sensitivity of the individual measures in relation to the construct (i.e. cognitive domain) that is being measured. [71]. Finally, only five of the 22 publications included in this review reported effect sizes for their statistical test outcomes [35, 40–42, 44]. Effect sizes are crucial for the interpretation of observed differences between groups. Even though *p*-values indicate whether or not a significant difference exists, they provide no information about the magnitude of the difference [72]. Moyle et al. [35] reported large effect sizes for observed deficits in cognitive performance in off-diet ET AwPKU. In contrast, reported deficits in cognitive functioning of ECT AwPKU in the PKU-COBESO study were small [40]. However, observed improvements in motor performance between T1 and T2 had medium to large effect sizes [42]. Furthermore, Jahja et al. [42] reported large effect sizes for differences in cognitive performance between ECT AwPKU with good versus poor metabolic control during childhood. Significant differences in performance on cognitive tasks between ET AwPKU and controls or normative or standardized data should be interpreted with caution, especially when no effect sizes have been reported. There is a need for greater homogeneity amongst measurement tools and the analysis and reporting of these in research in PKU.

#### Metabolic outcomes

Levels of metabolic control (i.e. Phe levels) at the time of testing varied both between and within study samples. A major contributor to such differences is the variation in guidelines for the management of PKU between countries and sometimes even between clinics within the same country. Furthermore, because guidelines have changed throughout the life of the ET AwPKU included in the research (e.g. diet for life is relatively recent advice and was probably introduced after some ET AwPKU included in the studies reviewed had already ceased the diet). Time of diagnosis, onset of treatment, and metabolic control throughout life are also likely to have varied amongst participants. Moreover, it has been shown that different methods for the analysis of dried blood spots (DBS) as well as differences in the size of the bloodspots that are measured could lead to significantly different results [73, 74], and oversaturation or undersaturation of the filter paper could lead to inaccurate results [74].

Research has also suggested that individuals with PKU often change adherence to their dietary management in the days leading up to a blood test, suggesting measured levels of Phe may underrepresent typical Phe levels [25, 75]. The large variance in Phe-data reported and limitations of measures of metabolic control, combined with relatively small sample sizes, reduces the likelihood that observed correlations are reliable. As a result of the variability in metabolic control between participants, several studies created subgroups of ET AwPKU with high or low Phe levels using different cut-off criteria. In addition to using different cut-off criteria to create subgroups for analysis, studies also differed in how they reported measures of metabolic control throughout life. Again, these discrepancies in reporting make it difficult to compare study outcomes and obtain a clear picture of how metabolic control throughout life influences cognition in ET AwPKU. Only a few of the studies included in this review explored the relationship between cognitive performance and Phe variation throughout life [17, 46, 47] and these found correlations across cognitive domains. Moreover, only one of the studies included measures of Phe:Tyr ratio but did not explore the relationship between this outcome and cognitive performance [48]. Limited research on the association between Phe:Tyr and EF in PKU suggests that high lifetime ratios rather than average Phe levels were associated with observed deficits in EF [76, 77]. It should be noted that Tyr levels obtained via DBS could be inaccurate if patients contaminate the filter paper by not washing their hands prior to blood sampling. Future research should include assessment of Phe fluctuations and Phe:Tyr ratio throughout life to enable a better understanding of the impact of metabolic control throughout life on outcomes in adulthood. However, due to limitations in measurements of metabolic control described previously, any observed associations should be interpreted with caution.

## Conclusions

Results from the studies included in this systematic review suggest that, despite early treatment, ET AwPKU have deficits in vigilance, WM, and motor skills compared to healthy controls. Long-term cognitive outcomes of ECT AwPKU remain unclear. Furthermore, several associations between cognitive performance and metabolic control throughout life were observed. However, these findings were inconsistent and therefore, it is difficult to determine the long-term effects of poor metabolic control at different stages in life on cognitive function in AwPKU.

To gain a better understanding of cognitive functioning and the development of cognitive deficits in ET AwPKU and ECT AwPKU future research would benefit from 1) (inter)national multicentre-studies; 2) more homogeneous samples; and 3) the inclusion of other nutritional measures that might influence cognitive functioning (e.g. Phe fluctuations, Phe:Tyr ratio and micronutrients, such as vitamin B12) and 4) attention to cognitive test selection and statistical analysis.

## Additional file

Additional file 1: **Table S1.** Summary of papers included within the systematic review. Data extraction table summarising the main characteristics of each of the papers included in this review. The table provides an overview of all information provided by each publication in regard to: the number of adults with phenylketonuria (PKU) and controls (where applicable), sex, age, IQ, classification of PKU, time of diagnosis, onset of treatment, treatment status, duration of treatment, measures of metabolic control and cognitive measures used and reported outcomes. (XLS 76 kb)

Additional file 2: **Table S2.** Overview of reported associations between metabolic control and measures of cognitive functioning in ET AwPKU. (DOCX 58 kb)

## Abbreviations

ADM: Attention Diagnostic Method
ANT: Amsterdam Neurological Tasks
AwPKU: Adults with Phenylketonuria
BBB: Blood Brain Barrier
BNT: Boston Naming Test
CANTAB: Cambridge Neuropsychological Test Automated Battery
COWAT: Controlled Oral Word Association Test
CPT: Conners’ Continuous Performance Task
CRT: Choice Reaction Time
CVLT: California Verbal Learning Test
DBS: Dried Blood Spot
D-KEFS: Delis-Kaplan Executive Function System
DPE: Dot Pattern Exercise
DSST: Digit Symbol (Substitution) Task
ECT AwPKU: Early and Continuously Treated Adults with Phenylketonuria
EF: Executive Functions
EPMT: Elithorn’s Perceptual Maze Test
ET AwPKU: Early Treated Adults with Phenylketonuria
ET: Early Treated
FI: Feature Integration task
FL: Flanker task
FPT: Faux-Pas Recognition Test
FR: Face Recognition task
FSIQ: Full Scale Intelligence Quotient
FSME: Finger Motor Speed Exercise
IDC: Index of Dietary Control
IFE: Identification of Facial Emotions test
IQ: Intelligence Quotient
LNAA: Large Neutral Amino Acids
LPE: Letter Pattern Exercise
MLS: Motorische Leistungsserie
MOT: Motor Screening Test
MS2D: Memory Search 2-Dimensions task
P&P: Pen and Paper
PAH: Phenylalanine Hydroxylase
Phe: Phenylalanine
Phe:Tyr (ratio): Ratio between levels of Phenylalanine and Tyrosine
PIQ: Performance Intelligence Quotient
PKU: Phenylketonuria
POI: Perceptual Organization Index
PPVT(−R): Peabody Picture Vocabulary Test(-Revised)
PRISMA: Preferred Reporting Items for Systematic Reviews and Meta-Analyses
PSI: Processing Speed Index
PU: Pursuit task
QATSDD: Quality Assessment Tool for Reviewing Studies with Diverse Design
RAVLT: Rey Auditory Verbal Learning test
RME: Reading the Mind in the Eyes test
ROCFT: Rey Österrieth Complex Figure Test
RVP: Rapid Visual Information Processing
SAD: Sustained Attention Dots
SOC: Stocking of Cambridge
SOPT: Self-Ordered Pointing Test
SRR: Systematic Research Review
SRT: Simple Reaction Time
SSP: Spatial Span
SST: Stop Signal Task
SSV: Set Shifting Visual task
STM: Short-Term Memory
SVAT: Sonneville Visual Attention Tasks (Precursor ANT)
SWM: Spatial Working Memory
TEA: Tests of Everyday Attention
TIQ: Total Intelligence Quotient
TMT: Trail Making Test
TMT-A: Trail Making Test part A
TMT-B: Trail Making Test part B
TOH: Tower of Hanoi
ToL: Tower of London
Trp: Tryptophan
Tyr: Tyrosine
VIQ: Verbal Intelligence Quotient
VSS: Visuo-Spatial Sequencing task
WAIS(−R): Wechsler Adult Intelligence Scale(–Revised)
WASI: Wechsler Adult Scale of Intelligence Scale
WCST: Wisconsin Card Sorting Test
WCST(-PR): Wisconsin Card Sorting Test(-*Perseverative Responses)*
WM: Working Memory
WMS: Wechsler Memory Scale
ZVT: Zahlen-Verbindungs-Test
