# Supplementary material for: A systematic review of cognitive functioning in early treated adults with phenylketonuria
Source: Orphanet J Rare Dis. 2018 Aug 30;13:150. doi: 10.1186/s13023-018-0893-4 (PMC6117942; doi:10.1186/s13023-018-0893-4)
Supplement: Supplementary file 2 — Table S2. Overview of reported associations between metabolic control and measures of cognitive functioning in ET AwPKU. (DOCX 58 kb) [file 13023_2018_893_MOESM2_ESM.docx]

**Additional file 2: Table S2 Overview of reported associations between metabolic control and measures of cognitive functioning in ET AwPKU.**

| **Attention And Processing Speed** | **Attentional Capacity** | | |  | | **Vigilance/Focus** | |  | |  | | **Processing Speed** |
| --- | --- | --- | --- | --- | --- | --- | --- | --- | --- | --- | --- | --- |
|  | Overall | Accuracy | Speed | | Overall | | Accuracy | | Speed | | Overall | |
| Concurrent Phe | **1/7^a^** | 0/1 | **1/1^a^** | | **1/3** | | 0/1 | | **1/1** | | **2/7^b^** | |
| Concurrent Tyr |  |  |  | |  | |  | |  | | 0/2 | |
| Recent Phe | 0/1 | 0/1 | 0/1 | | 0/1 | |  | |  | |  | |
| **CHILDHOOD Phe** |  |  |  | |  | |  | |  | |  | |
| 0-10 years |  |  |  | |  | |  | |  | |  | |
| *Average* | **1/1** |  |  | | 0/1 | |  | |  | |  | |
| *Variation* | **1/1** |  |  | | 0/1 | |  | |  | |  | |
| *Average + variation* | 0/1 |  |  | | 0/1 | |  | |  | |  | |
| 0-12 years | 1/2 |  |  | |  | | 0/1 | | 0/1 | |  | |
| 0-4/1-4 years | 0/1 | 0/1 | **1/1** | | 0/1 | |  | |  | |  | |
| 5.5-6 years (median) | **1/4** |  |  | | 0/1 | |  | |  | | 0/2 | |
| 5-8 years | 0/1 | 0/1 | **1/1^a^** | | 0/1 | |  | |  | |  | |
| 9.5-10 years (median) | 0/4 |  |  | | 0/1 | |  | |  | | 0/2 | |
| 9-12/10-12 years | 0/1 | 0/1 | 0/1 | | **1/2** | |  | |  | | **1/1** | |
| **ADOLESCENT Phe** |  |  |  | |  | |  | |  | |  | |
| 11-16 years |  |  |  | |  | |  | |  | |  | |
| *Average* | 0/1 |  |  | | 0/1 | |  | |  | |  | |
| *Variation* | 0/1 |  |  | | 0/1 | |  | |  | |  | |
| *Average + variation* | 0/1 |  |  | | 0/1 | |  | |  | |  | |
| 12 years – adulthood | 0/1 |  |  | |  | |  | |  | |  | |
| 13-15/13-16/13-17 years | 0/1 | 0/1 | 0/1 | | **1/2** | |  | |  | | **1/1** | |
| 16-18 years | 0/1 | 0/1 | 0/1 | | **1/2** | |  | |  | | **1/1** | |
|  |  |  |  | |  | |  | |  | |  | |
|  | Overall | Accuracy | Speed | | Overall | | Accuracy | | Speed | | Overall | |
| **ADULT Phe** |  |  |  | |  | |  | |  | |  | |
| 17+ years |  |  |  | |  | |  | |  | |  | |
| *Average* | **1/1** |  |  | | **1/1** | |  | |  | |  | |
| *Variation* | 0/1 |  |  | | **1/1** | |  | |  | |  | |
| *Average + variation* | **1/1** |  |  | | **1/1** | |  | |  | |  | |
| 17-20/19-21 years | 0/2 | 0/2 | 0/2 | | **1/3** | |  | |  | | **1/1** | |
| 21-24 years | 0/1 | 0/1 | 0/1 | | 0/1 | |  | |  | |  | |
| 25-28 years | **1/1** | 0/1 | 0/1 | | **1/1** | |  | |  | |  | |
| 29-32 years |  | 0/1 | 0/1 | |  | |  | |  | |  | |
| **LIFETIME Phe** |  |  |  | |  | |  | |  | |  | |
| Lifetime Phe |  |  |  | |  | | 0/1 | | 0/1 | | 0/3 | |
| *Average* | **1/1** |  |  | | 0/1 | |  | |  | |  | |
| *Variation* | 0/1 |  |  | | **1/1** | |  | |  | |  | |
| *Average + variation* | **1/1** |  |  | | **1/1** | |  | |  | |  | |
| 0 years – adulthood | 0/1 |  |  | |  | |  | |  | |  | |

| **Executive Functions** | **Complex Executive Functions** | | | | **Inhibition** | | | **Working Memory** | | | | |
| --- | --- | --- | --- | --- | --- | --- | --- | --- | --- | --- | --- | --- |
|  | Overall | Accuracy | Speed | Overall | | Accuracy | Speed | Overall | | Accuracy | | Speed |
| Concurrent Phe | **2/7^a^** | 0/2 | 0/2 | 0/1 | | 0/1 | **1/3** | **1/5** | | 0/9 | | **3/7** |
| Concurrent Tyr | 0/1 | 0/1 | 0/1 |  | |  |  |  | | 0/1 | |  |
| Recent Phe |  |  |  |  | |  | 0/1 | **1/1^a^** | |  | |  |
| **CHILDHOOD Phe** |  |  |  |  | |  |  |  | |  | |  |
| 0-10 years |  |  |  |  | |  |  |  | |  | |  |
|  |  |  |  |  | |  |  |  | |  | |  |
|  | Overall | Accuracy | Speed | Overall | | Accuracy | Speed | Overall | | Accuracy | | Speed |
| **CHILDHOOD Phe (continued)** |  |  |  |  | |  |  |  | |  | |  |
| *Average* | 0/1 |  |  | 0/1 | |  |  | 0/1 | |  | |  |
| *Variation* | **1/1** |  |  | 0/1 | |  |  | 0/1 | |  | |  |
| *Average + variation* | 0/1 |  |  | 0/1 | |  |  | 0/1 | |  | |  |
| 0-12 years |  | 0/1 | **1/1** |  | | 0/2 | **1/2** |  | | 0/6 | | 0/5 |
| 0-4/1-4 years | 0/3 |  |  |  | |  | 0/1 | 0/1 | | 0/2 | | 0/2 |
| 5.5-6 years (median) | 0/1 |  |  |  | |  |  | **1/1** | |  | |  |
| 5-8 years |  |  |  |  | |  | 0/1 | 0/1 | | 0/2 | | 0/2 |
| 9.5-10 years (median) | 0/1 |  |  |  | |  |  | 0/1 | |  | |  |
| 9-12/10-12 years |  |  |  |  | |  | 0/1 | 0/1 | | 0/2 | | 0/2 |
| **ADOLESCENT Phe** |  |  |  |  | |  |  |  | |  | |  |
| 11-16 years |  |  |  |  | |  |  |  | |  | |  |
| *Average* | **1/1** |  |  | 0/1 | |  |  | 0/1 | |  | |  |
| *Variation* | **1/1** |  |  | 0/1 | |  |  | 0/1 | |  | |  |
| *Average + variation* | **1/1** |  |  | 0/1 | |  |  | 0/1 | |  | |  |
| 13-15/13-16/13-17 years |  | **1/1** | 0/1 |  | | **1/1** | 0/2 | 0/1 | | 0/2 | | **1/2** |
| 16-18 years |  |  |  |  | |  | 0/1 |  | |  | |  |
| **ADULT Phe** |  |  |  |  | |  |  |  | |  | |  |
| 17+/18+ years |  | 0/1 | 0/1 |  | | 0/1 | 0/1 |  | |  | |  |
| *Average* | 0/1 |  |  | 0/1 | |  |  | 0/1 | |  | |  |
| *Variation* | 0/1 |  |  | 0/1 | |  |  | 0/1 | |  | |  |
| *Average + variation* | **1/1** |  |  | 0/1 | |  |  | 0/1 | |  | |  |
|  |  |  |  |  | |  |  |  | |  | |  |
|  |  |  |  |  | |  |  |  | |  | |  |
|  |  |  |  |  | |  |  |  | |  | |  |
|  | Overall | Accuracy | Speed | Overall | | Accuracy | Speed | Overall | | Accuracy | | Speed |
| **ADULT Phe (continued)** |  |  |  |  | |  |  |  | |  | |  |
| 17-20/19-21 years |  |  |  |  | |  | 0/1 | 0/1 | | 0/2 | | 0/2 |
| 21-24 years |  |  |  |  | |  | 0/1 | **1/1** | | 0/2 | | 0/2 |
| 25-28 years |  |  |  |  | |  | 0/1 | **1/1** | | 0/2 | | 0/2 |
| 29-32 years |  |  |  |  | |  | 0/1 |  | | 0/2 | | 0/2 |
| **LIFETIME Phe** |  |  |  |  | |  |  |  | |  | |  |
| Lifetime Phe | 0/1 | 0/1 | 0/1 |  | | 0/1 | 0/1 | 0/2 | | **1/8** | | 0/5 |
| *Average* | 0/1 |  |  | 0/1 | |  |  |  | |  | |  |
| *Variation* | **1/1** |  |  | 0/1 | |  |  |  | |  | |  |
| *Average + variation* | **1/1** |  |  | 0/1 | |  |  |  | |  | |  |
| **MULTIPLE TESTING (Phe)** |  |  |  |  | |  |  |  | |  | |  |
| IDC1 |  | 0/1 | **1/1** |  | | 0/1 | 0/1 |  | |  | |  |
| IDC2 |  | 0/1 | 0/1 | **1/1** | |  | 0/1 |  | |  | |  |
| IDC difference (IDC2-IDC1) |  | 0/1 | 0/1 |  | | 1/1 | 0/1 |  | |  | |  |
| IDC0-1 (8-13 years) | 0/3 |  |  |  | |  |  |  | |  | |  |
| IDC1-2 | 2/3 |  |  |  | |  |  |  | |  | |  |
| IDC0-2 (22-27 years) | 1/3 |  |  |  | |  |  |  |  | |  | |

| **Executive Functions (EF)**  **+ Language** | **Verbal Fluency (EF)** | **Basic Language Skills:**  **Semantic Processing** | **Complex Language Skills** | **Orthographic Language** | **Spoken Language** |
| --- | --- | --- | --- | --- | --- |
| Concurrent Phe | **1/5** | **2/11** | **1/24** | 0/1 | 0/1 |
| Recent Phe | 0/1 |  |  |  |  |
|  |  |  |  |  |  |
| **CHILDHOOD Phe** |  |  |  |  |  |
| 0-10 years |  |  |  |  |  |
| *Average* |  | 0/9 | 0/21 | 0/1 | 0/1 |
| *Variation* |  | 0/9 | 0/21 | 0/1 | **1/1** |
| *Average + variation* |  |  |  | 0/1 | 0/1 |
| 0-12 years |  |  |  |  |  |
| 0-4/1-4 years | 0/1 |  |  |  |  |
| 5.5-6 years (median) | 0/1 | **2/2** | **1/2** |  | **4/5** |
| 5-8 years | 0/1 |  |  |  |  |
| 9.5-10 years (median) | **1/2** | **2/2** | **1/2** |  | **4/5** |
| 9-12/10-12 years | 0/1 |  |  |  |  |
| **ADOLESCENT Phe** |  |  |  |  |  |
| 11-16 years |  |  |  |  |  |
| *Average* |  | 0/9 | 0/21 | 0/1 | 0/1 |
| *Variation* |  | 0/9 | 0/21 | 0/1 | **1/1** |
| *Average + variation* |  |  |  | 0/1 | **1/1** |
| 13-15/13-16 /13-17 years | 0/1 |  |  |  |  |
| 16-18 years | 0/1 |  |  |  |  |
| **ADULT Phe** |  |  |  |  |  |
| 17+/18+ years |  |  |  |  |  |
| *Average* |  | 0/9 | 0/21 | 0/1 | 0/1 |
| *Variation* |  | 0/9 | 0/21 | 0/1 | **1/1** |
| *Average + variation* |  |  |  | 0/1 | **1/1** |
|  |  |  |  |  |  |
|  |  |  |  |  |  |
|  | Overall | Overall | Overall | Overall | Overall |
| **ADULT Phe (continued)** |  |  |  |  |  |
| 17-20/19-21 years | 0/1 |  |  |  |  |
| 21-24 years | 0/1 |  |  |  |  |
| 25-28 years | 0/1 |  |  |  |  |
| **LIFETIME Phe** |  |  |  |  |  |
| Lifetime Phe | 0/1 |  | 0/1 | 0/1 | 0/1 |
| *Average* |  | 0/9 | 0/21 | 0/1 | **1/1** |
| *Variation* |  | 0/9 | 0/21 | 0/1 | **1/1** |
| *Average + variation* |  |  |  | 0/1 | 0/1 |

| **Memory and Learning (M&L)** | **GM^c^** | **Immediate Recall**  **(Verbal/Visual)** | | | | | **Delayed Recall (Verbal/Visual)** | | | | **Recognition**  **(Verbal/Visual)** | | | **Verbal M&L** | **Visual M&L** |
| --- | --- | --- | --- | --- | --- | --- | --- | --- | --- | --- | --- | --- | --- | --- | --- |
|  |  | | Overall | Verbal | Visual | Overall | | Verbal | Visual | Overall | | Verbal | Visual | Overall | Overall |
| Concurrent Phe | **1/1** | | **1/1** | **1/2** | **1/2** |  | | 0/3 | **1/2** |  | | **1/2** |  | **1/1** | **1/1** |
| **CHILDHOOD Phe** |  | |  |  |  |  | |  |  |  | |  |  |  |  |
| 0-10 years |  | |  |  |  |  | |  |  |  | |  |  |  |  |
| *Average* |  | |  |  |  |  | |  |  |  | |  |  | **1/1** | **1/1** |
| *Variation* |  | |  |  |  |  | |  |  |  | |  |  | 0/1 | **1/1** |
| *Average + variation* |  | |  |  |  |  | |  |  |  | |  |  | 0/1 | 0/1 |
| 0-4/1-4 years |  | |  |  | 0/1 |  | |  |  |  | |  |  |  |  |
| 5.5-6 years (median) |  | |  | **1/1** |  |  | | 0/2 | **1/1** |  | | **1/1** |  |  |  |
| 9.5-10 years (median) |  | |  | 0/1 |  |  | | 0/2 | 0/1 |  | | 0/1 |  |  |  |
|  |  | |  |  |  |  | |  |  |  | |  |  |  |  |
|  |  | | Overall | Verbal | Visual | Overall | | Verbal | Visual | Overall | | Verbal | Visual | Overall | Overall |
| **ADOLESCENT Phe** |  | |  |  |  |  | |  |  |  | |  |  |  |  |
| 11-16 years |  | |  |  |  |  | |  |  |  | |  |  |  |  |
| *Average* |  | |  |  |  |  | |  |  |  | |  |  | 0/1 | **1/1** |
| *Variation* |  | |  |  |  |  | |  |  |  | |  |  | **1/1** | **1/1** |
| *Average + variation* |  | |  |  |  |  | |  |  |  | |  |  | **1/1** | **1/1** |
| **ADULT Phe** |  | |  |  |  |  | |  |  |  | |  |  |  |  |
| 17+/18+ years |  | |  |  |  |  | |  |  |  | |  |  |  |  |
| *Average* |  | |  |  |  |  | |  |  |  | |  |  | **1/1** | **1/1** |
| *Variation* |  | |  |  |  |  | |  |  |  | |  |  | 0/1 | **1/1** |
| *Average + variation* |  | |  |  |  |  | |  |  |  | |  |  | **1/1** | **1/1** |
| **LIFETIME Phe** |  | |  |  |  |  | |  |  |  | |  |  |  |  |
| Lifetime Phe |  | | 0/1 | 0/1 | 0/1 |  | | 0/1 | 0/1 |  | | 0/1 |  |  |  |
| *Average* |  | |  |  |  |  | |  |  |  | |  |  | 0/1 | **1/1** |
| *Variation* |  | |  |  |  |  | |  |  |  | |  |  | **1/1** | **1/1** |
| *Average + variation* |  | |  |  |  |  | |  |  |  | |  |  | **1/1** | **1/1** |
| **MULTIPLE TESTING (Phe)** |  | |  |  |  |  | |  |  |  | |  |  |  |  |
| IDC0-1 (8-13 years) |  | |  |  | 0/1 |  | |  |  |  | |  |  |  |  |
| IDC1-2 |  | |  |  | 0/1 |  | |  |  |  | |  |  |  |  |
| IDC0-2 (22-27 years) |  | |  |  | 0/1 |  | |  |  |  | |  |  |  |  |

| **Various Cognitive Domains** | **Motor** | | | **Social-Cognitive Abilities** | | | **Visual-Perceptual Abilities** | |
| --- | --- | --- | --- | --- | --- | --- | --- | --- |
|  | Overall | Accuracy/  Stability | Overall | | Accuracy | Speed | | Overall |
| Concurrent Phe | **1/7** | 0/3 | 0/2 | | 0/2 | 0/2 | | 0/6 |
| Concurrent Tyr |  | 0/1 |  | |  |  | |  |
| **CHILDHOOD Phe** |  |  |  | |  |  | |  |
| 0-10 years |  |  |  | |  |  | |  |
| Average | 0/1 |  |  | |  |  | |  |
| Variation | **1/1** |  |  | |  |  | |  |
| Average + variation | 0/1 |  |  | |  |  | |  |
| 0-12 years | 0/4 | **2/2** |  | |  |  | |  |
| 0-4/1-4 years |  |  |  | |  |  | | 0/1 |
| 5.5-6 years (median) | 0/1 |  |  | |  |  | | 0/4 |
| 5-8 years |  |  | 0/2 | | 0/2 | 0/2 | |  |
| 9.5-10 years (median) | 0/1 |  |  | |  |  | | 0/4 |
| 9-12/10-12 years |  |  | 0/2 | | 0/2 | 0/2 | |  |
| **ADOLESCENT Phe** |  |  |  | |  |  | |  |
| 11-16 years |  |  |  | |  |  | |  |
| Average | **1/1** |  |  | |  |  | |  |
| Variation | **1/1** |  |  | |  |  | |  |
| Average + variation | **1/1** |  |  | |  |  | |  |
| 13-15/13-16/13-17 years |  | 0/2 | 0/2 | | 0/2 | 0/2 | |  |
| 12 years – adulthood | 0/4 |  |  | |  |  | |  |
| **ADULT Phe** |  |  |  | |  |  | |  |
| 17+ / 18+ years |  | 0/2 | 0/2 | | 0/2 | 0/2 | |  |
| *Average* | **1/1** |  |  | |  |  | |  |
| *Variation* | **1/1** |  |  | |  |  | |  |
| *Average + variation* | **1/1** |  |  | |  |  | |  |
|  | Overall | Accuracy/  Stability | Overall | | Accuracy | Speed | | Overall |
| **LIFETIME Phe** |  |  |  | |  |  | |  |
| Lifetime Phe |  | 0/1 |  | |  |  | |  |
| *Average* | **1/1** |  |  | |  |  | |  |
| *Variation* | **1/1** |  |  | |  |  | |  |
| *Average + variation* | **1/1** |  |  | |  |  | |  |
| 0 years – adulthood | 0/4 |  |  | |  |  | |  |
| **MULTIPLE TESTING** |  |  |  | |  |  | |  |
| IDC1 |  | **2/2** |  | |  |  | |  |
| IDC2 |  | 0/2 |  | |  |  | |  |
| IDC difference (IDC2-IDC1) |  | 0/2 |  | |  |  | |  |
| IDC0-1 (8-13 years) |  |  |  | |  |  | | 0/1 |
| IDC1-2 |  |  |  | |  |  | | 0/1 |
| IDC0-2(22-27 years) |  |  |  | |  |  | | 0/1 |

*^a^ correlations not in the expected direction: lower Phe associated with worse cognitive performance; ^b^ not all correlations in the expected direction (see ^a^); ^c^ GM = General Memory*
